# Thermal time constant: optimising the skin temperature predictive modelling in lower limb prostheses using Gaussian processes

**DOI:** 10.1049/htl.2015.0023

**Published:** 2016-05-20

**Authors:** Neha Mathur, Ivan Glesk, Arjan Buis

**Affiliations:** 1 Department of Electronic and Electrical Engineering, University of Strathclyde, 204 George Street, Glasgow G1 1XW, UK; 2 Department of Biomedical Engineering, University of Strathclyde, Glasgow G4 0NW, UK

**Keywords:** biothermics, prosthetics, skin, thermal time constant, skin temperature predictive modelling, lower limb prostheses, Gaussian processes, body-device interface, tissue health, heat dissipation, hard socket, residual limb temperature, perspiration

## Abstract

Elevated skin temperature at the body/device interface of lower-limb prostheses is one of the major factors that affect tissue health. The heat dissipation in prosthetic sockets is greatly influenced by the thermal conductive properties of the hard socket and liner material employed. However, monitoring of the interface temperature at skin level in lower-limb prosthesis is notoriously complicated. This is due to the flexible nature of the interface liners used which requires consistent positioning of sensors during donning and doffing. Predicting the residual limb temperature by monitoring the temperature between socket and liner rather than skin and liner could be an important step in alleviating complaints on increased temperature and perspiration in prosthetic sockets. To predict the residual limb temperature, a machine learning algorithm – Gaussian processes is employed, which utilizes the thermal time constant values of commonly used socket and liner materials. This Letter highlights the relevance of thermal time constant of prosthetic materials in Gaussian processes technique which would be useful in addressing the challenge of non-invasively monitoring the residual limb skin temperature. With the introduction of thermal time constant, the model can be optimised and generalised for a given prosthetic setup, thereby making the predictions more reliable.

## 1 Introduction

Increased heat and perspiration is a common complaint of many amputees. Hagberg and Branemark [1] found that a significant percentage (72%) of their study population (transfemoral amputees, *n* = 95) reported the problem of heat/sweating as their most common complaint. This was followed by skin irritation caused by the prosthesis, which was considered moderate or worst in 62% of the group. Hoaglund *et al.* [2] discovered similar complaints with 70% of their study population (veteran amputees, *n* = 174) reporting perspiration problems. To compound the problem of sweating further, it has also been documented that a small amount of sweat on the skin will increase the frictional forces that exist between the body/device interface [3], making it more susceptible to breakdown [4]. Skin breakdown is a major issue for amputees and if good skin condition is not maintained the device may not be worn [5].

Further to this the moist and warm environment of a prosthetic socket promotes maceration of the skin, which in turn may lead to invasion of hair follicles by bacteria [6]. The skin plays a major role in thermoregulation of the body via radiation of heat. The properties of prosthetic sockets create an environment, where this heat transfer is influenced by the insulating properties of commonly used socket materials and liners. These materials inhibit the body's ability to radiate heat effectively [7] and may be a cause of the reported thermal discomfort mentioned early. Although the mechanical properties of these materials have been well documented [8–10] less is known about how these materials transfer heat [7, 11, 12]. Before the problem of thermal discomfort can be tackled, further investigation into the thermal properties of prosthetic materials is first required. This will assist in the further understanding of prosthetic materials and enable clinicians to identify the materials which are the least effective in transferring the heat radiating from the human body to the outside environment.

A mathematical model using the Gaussian processes for machine learning (GPML) to predict the stump skin temperature of the amputee by measuring the in-socket (liner) temperature has been developed [13]. This is a supervised learning algorithm in which the hyperparameters and the covariance matrix of the Gaussian process model harness the experimental data for training and prediction. The residual limb skin temperature and its corresponding liner temperature greatly depend on the thermal properties of the prosthetic materials in use. The thermal conductivities of different liner and socket materials have been investigated by Klute *et al*. [7]. They assessed single layers of prosthetic socket material and found that thermoplastic and carbon fibre socket materials have very similar thermal conductivities. The above study investigated only individual layers of socket and liner materials. However, prosthetic sockets are composed of two, sometimes three layers of differing materials and there is a need to define the effect of the thermal properties of these layers in combination. This Letter addresses it and in our experiments the thermal time constant of single layers of materials was first investigated, and then combined those materials in various combinations to give a more realistic representation of a prosthetic socket.

The advantage of evaluating thermal time constant of the prosthetic materials over any other thermal properties such as thermal conductivity, specific heat or heat transfer coefficient is the simplicity in its measurement and calculation and also implementing it in the previously designed Gaussian model. By introducing the thermal time constant value in the covariance function of the Gaussian model, the model can be optimised and generalised for lower-limb prosthetic users with a similar prosthetic setup. Also, the accuracy of the model is improved from ±0.8 to ±0.5°C. This would especially be useful in addressing the challenge of non-invasively monitoring the residual limb skin temperature for a wider amputee population.

## 2 Methodology – temperature measurement

According to the law of thermodynamics, heat transfer *F*, from the heat source to the test material at a given time is proportional to the difference in temperature between the heat source and test material

(1) $$ - F = h{A_{\rm s}}\left({T\left(t \right)- {T_{\rm h}}} \right)\eqno\lpar 1\rpar $$

where *h* is the heat transfer coefficient, *A*_s_ is the surface area, *T*(*t*) is the temperature of the test material at time *t* and *T*_h_ is the constant temperature of the heat source. The addition of heat leads to the rise in temperature of test material which is given by

(2) $$\rho {c_p}V\left({\displaystyle{{{\rm d}T} \over {{\rm d}t}}} \right)= F\eqno\lpar 2\rpar $$

where *ρ* is the density, *c*_*p*_ is the specific heat and *V* is the volume of the test material. Equating these two equations for heat transfer

(3) $$\rho {c_p}V\left({\displaystyle{{{\rm d}T} \over {{\rm d}t}}} \right)= \; - h{A_{\rm s}}\left({T\left(t \right)- {T_{\rm h}}} \right)\eqno\lpar 3\rpar $$

This can be further rewritten as

(4) $$\displaystyle{{\; \; {\rm d}T} \over {{\rm d}t}} = \displaystyle{1 \over \tau }\left({{T_{\rm h}} - T} \right)\eqno\lpar 4\rpar $$

Here the time constant *τ* can be defined as

(5) $$\tau = \displaystyle{{\rho {c_p}V} \over {h{A_{\rm s}}}}\eqno\lpar 5\rpar $$

This implies that the time constant is indicative of temperature response of the material. When the temperature of the heat source is constant, the rate of change of test material temperature is given by

(6) $$\displaystyle{{{\rm d\Delta }T} \over {{\rm d}t}} = - \, \displaystyle{1 \over \tau }{\rm \Delta }T\; \eqno\lpar 6\rpar $$

where Δ*T* = *T* − *T*_h_.

Solving this equation gives the difference between the temperature of the test material and the heat source Δ*T* as a function of time *t*

(7) $$\Delta T\lpar t\rpar = \Delta {T_0}{{\rm e}^{ - {t / \tau }}}\eqno\lpar 7\rpar $$

where Δ*T*_0_ is the initial temperature difference between the temperature, at time *t* = 0 .This indicates that the rate at which the temperature of the test material approaches the heat source temperature slows exponentially. Thus, the time constant that is derived from the principles of heat transfer provides a much simpler method to envision the thermal behaviour of a material. To measure the degree of thermal responsiveness of the prosthetic material, the thermal time constant *τ* is evaluated which is defined as the time required for the material at a certain temperature to reach 63.2% of the specified final temperature.

To explore the thermal properties of these materials when used individually and in combination, a number of liner and socket materials of dimension 100 mm × 100 mm were selected to provide a range representing those commonly used by lower-limb amputees. Table 1 indicates materials and thickness of the socket and liner specimens used in this Letter. The experimental setup included a heat source (heating tape Omega Engineering: 13 mm × 1.22 m, 312 W, 240 V) whose temperature could be controlled through a proportional–integral–derivative (PID) controller. The other equipments used were solid state relay (SSR) to provide safety to the circuit; ten-pin terminal block to allow connection of all devices; K-type thermocouples and a four-channel thermocouple thermometer. The idea is to duplicate the cross-section of the prosthesis by arranging the heating tape (which would be maintained at a steady temperature by the PID controller and would be emulating a section of residual limb of the amputee), liner and socket materials on top of each other.

**Table 1:** Socket and liner materials used for the study

Name	Material	Thickness, mm
Alpha Locking (liner)	co-polymer	6
Iceross Comfort (liner)	silicone	6
Iceross Original (liner)	silicone	3
OttoBock Technogel (liner)	polyurethane	6
Pe-lite (liner)	closed cell foam	5
stump sock	terry	0.7
thermoplastic (socket material)	co-polymer polypropylene	4.7
thermosetting lay-up (socket material)	compound of materials	4
carbon fibre lay-up (socket material)	compound of materials	4.8

**Fig. 1:**
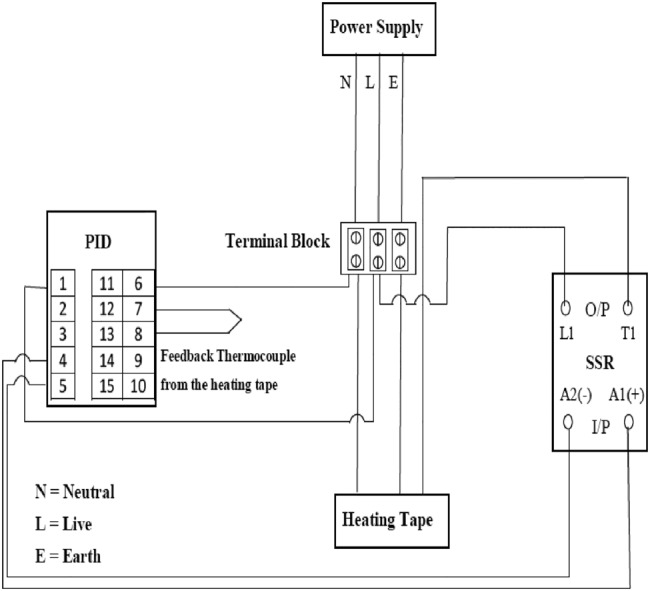
Schematic of the experimental setup utilised for measuring the thermal time constant of prosthetic materials

The heat source was the heating tape which lay flat on a 15 cm × 15 cm sheet of aluminium with an identical sized sheet of aluminium then placed on top of the heating tape forming a sandwich. The two sheets of aluminium were secured to each other by string from the heating tape. This circuit also incorporated an SSR and a terminal block. The SSR was used as a switch in the circuit receiving a small input voltage from the PID controller and controlling a larger output voltage of the heating source. The terminal block was required to make all the connections possible. The temperature of the heating tape was measured using a type K thermocouple that was also connected to the PID controller. This thermocouple provided feedback to the PID controller of the temperature on its surface, and the controller could make the necessary adjustments to the system to get the desired heating tape temperature. Fig. 1 indicates the schematic of the experimental setup described above.

To determine reliability of the experimental setup, the system was switched on and a set point of 29°C was selected. The experimental setup reached this temperature steadily and held it there successfully. Also, when the temperature was increased by 1–30°C, the circuit increased the temperature and also held it at the new set point of 30°C. This process was repeated, increasing by 1°C until 40°C was reached. From this process, it was decided that the heating tape and PID controller provide sufficient control of the temperature for the experiment to proceed.

## 3 Experimental process

**Fig. 2:**
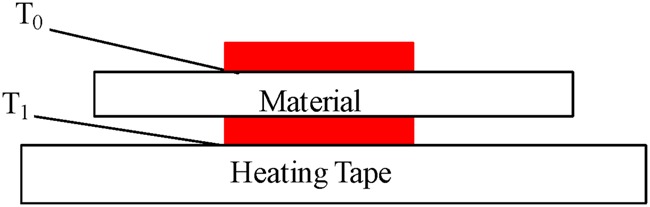
Schematic illustrating the placement of the prosthetic material (either liner or socket) on the heating tape. Interface temperatures T_1_ and T_0_ are measured by thermocouples

The heating tape was to be heated to 30°C and the circuit given sufficient time to come to rest. The temperature of the heating tape *T*_1_ was measured using one of the type *K* thermocouples. Full contact of the thermocouple was ensured by using polyimide adhesive tape labels (rated to 100°C). All data was collected on a computer connected to the thermocouple data logger and analysed using software provided with it. The prosthetic materials (liner and socket) were first tested individually to study their thermal behaviour in terms of the time constant. Along with the thermocouple on the heating tape, a second thermocouple was placed on the outer surface of the test material to measure the temperature at this point *T*_0_. Fig. 2 is a diagrammatic representation of this setup. Recording began at 30°C and only stopped when *T*_1_ = *T*_0_ or *T*_0_ had come to a steady temperature. The material was removed from the heating tape and allowed to cool to room temperature. Simultaneously the temperature of the heating tape was increased by 2°C and allowed to reach a steady temperature. The material was placed back on the heating source and recording began again. This process was repeated for increasing values of *T*_1_ by 2°C until 40°C was reached. Data collected by the thermocouple data logger and software was the temperature of the heating tape *T*_1_, temperature of the outer surface of the prosthetic material under test *T*_0_ and length of experiment (time). This routine was repeated until all materials (as in Table 1) had been tested individually. Once all materials had been individually tested, the next stage was to measure the temperature profile of the liner and socket material when used together. This is done by placing the socket material on top of the liner material and then putting this stack of materials on the heating tape.

**Fig. 3:**
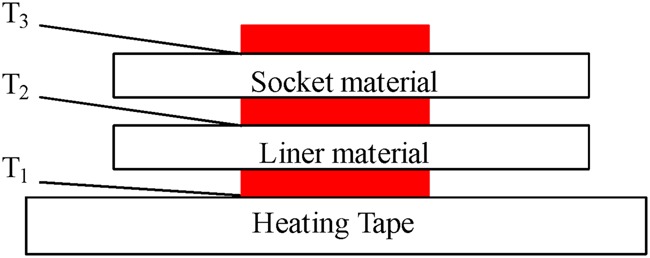
Schematic illustrating the placement of the liner and socket material on the heating tape. Interface temperatures T_1_, T_2_ and T_3_ are measured by thermocouples

Similar to the testing individual materials, thermocouples were placed on the outer surface of the each of materials – liner and socket. Fig. 3 shows a diagrammatic representation for testing of these two stacked materials. Data collection was only stopped this time when *T*_1_ = *T*_3_ or *T*_3_ ceased to increase in temperature. Data collected was temperature of the heating tape *T*_1_, temperature of the outer surface of the liner material *T*_2_, temperature of the outer surface of the socket material *T*_3_ and duration of experiment. A number of two-layer prosthetic material combinations were tested. Measurement followed the same procedure as described above, i.e. beginning at 30°C and rising by 2°C until 40°C, then the materials were changed and measurement was repeated again for combinations listed in Table 3. All the experimentation were done in an ambient temperature of 22°C.

When the prosthetic materials were tested individually, it was noted that as the set point temperature of the heating tape *T*_1_ was increased, the maximum temperature reached by the material *T*_0_ increased and so did the difference between the set point temperature and material temperature *T*_1_ − *T*_0_. What this indicates is that with increasing temperature *T*_1_, more heat is transferred through the material, but there is also an increase in the amount of heat lost in this process. Similarly, it is observed that when liner-socket materials are in tested together as the set point temperature of the heating tape was increased *T*_1_, the maximum temperature reached by the liner material *T*_2_ and socket material *T*_3_ increased and so did the difference between the set point temperature and material temperatures: namely, *T*_1_ − *T*_2_ and *T*_1_ − *T*_3_. Also, it takes them longer to reach a steady temperature than when they were tested individually. This indicates that the rate of heat transfer decreases when prosthetic materials are used in combinations.

## 4 Determination of thermal time constant

The thermal profile of the prosthetic materials when tested individually or in combination with another material was obtained from the thermocouple data logger. The results indicated that the heat transfers through prosthetic materials in a logarithmic fashion – initially with a fast rate of heat transfer which decreases as time increases – and reaches a steady state at a temperature lower than that of the heating tape temperature.

**Fig. 4:**
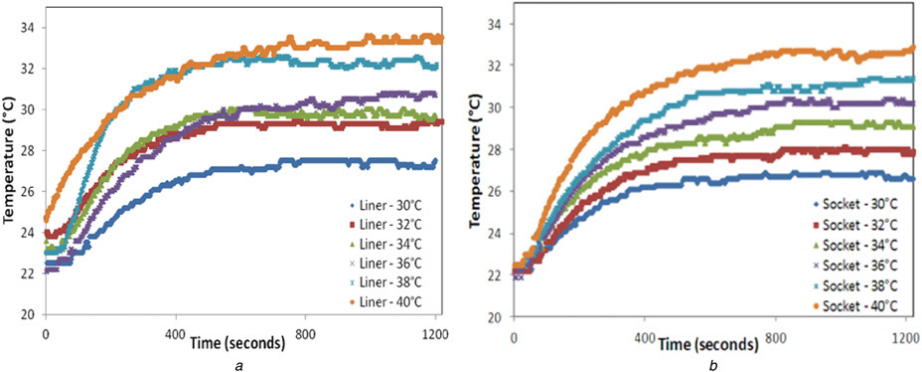
Temperature profile of *a* Polyurethane liner *b* Thermosetting socket material at different heat source temperatures when tested individually using the experimental setup

**Fig. 5:**
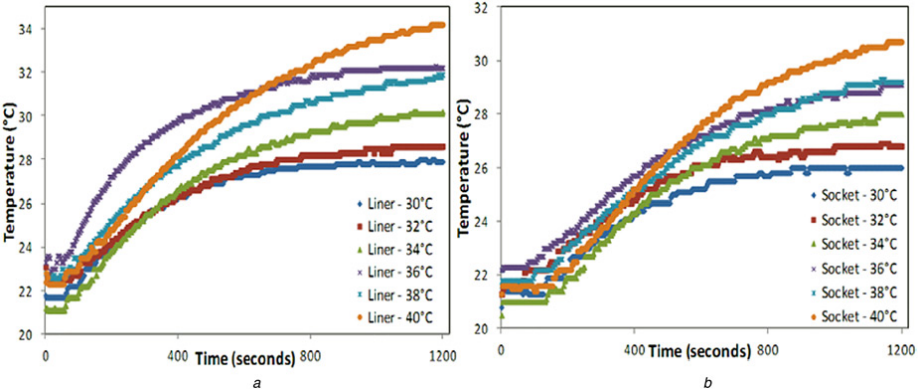
Temperature profile of *a* Polyurethane liner *b* Thermosetting socket material at different heat source temperatures when tested in combination (by being placed on top of the other) using the experimental setup

Of all the two layer combinations that were tested, the combination that was of most interest was the 6 mm thick polyurethane liner along with 4 mm thick thermosetting lay-up socket material as this is the most widely used liner-socket pair in practice. The thermal graphs recorded for the above-mentioned materials when used individually and in combination are indicated in Figs. 4 and 5. Fig. 4 indicates the temperature profile of the polyurethane liner and thermosetting material when tested individually using the lay-up shown in Fig. 2. It can be seen that the interface temperatures *T*_0_ when plotted against time follow a logarithmic profile and reaching steady state in the end. When the above-mentioned liner-socket combination was tested together using lay-up shown in Fig. 3, the thermal responses *T*_2_ and *T*_3_ are indicated in Figs. 5*a* and *b*, respectively. From these graphs, it can be seen that the thermal response of the materials is slower when they are used in combination than when they are used individually. These graphs were then used to calculate the thermal time constant of the prosthetic materials which would indicate how quickly the heat flows from the source to the opposite end of the material.

Utilising this behaviour of the prosthetic materials, the thermal time constant *τ* is computed using the logarithmic method. Time constants are parameters of systems that obey first order, linear differential equations. Consider that the equation for the thermal response curve of the prosthetic test material is

(8) $$x\left(t \right)= x\lpar 0\rpar {{\rm e}^{ - {t / \tau }}}\; \eqno\lpar 8\rpar $$

where *x*(*t*) is the temperature of the test material at ambient time *t*, *x*(0) being the initial temperature response and *τ* being the thermal time constant indicating how quick is the system response. Taking the natural log of the response curve given by (8) we have

(9) $${\rm ln}\left[{x\left(t \right)} \right]= \ln \left[{x\left(0 \right)} \right]- \, \displaystyle{t \over \tau }\eqno\lpar 9\rpar $$

Equation (9) can be thought of as a straight line with the thermal response plotted against time. This implies that if the temperature of the test material is plotted with respect to time, then the slope of the line is the time constant and the intercept is the natural log of initial value. Using this concept, the temperature profile of the material when tested individually or in combination with another material as recorded by the data logger, can be utilised to compute the thermal time constant. The steps below detail the logarithmic method technique used to determine the thermal time constant when a prosthetic material is tested individually.

- The steady-state temperature of the material *T*_0ss_ is determined.
- The temperature at ambient time *T*_0_ is subtracted from the steady-state value so that an exponentially decaying dataset is created.
- The natural log of the exponentially decaying data as computed in step 2 ln[*x*(*T*_0ss_) − *x*(*T*_0_)] is taken and plotted with respect to time. Using regression, a line of best fit is generated and the slope is computed. The slope is a measure of the thermal time constant.

Similar procedure is adopted when a prosthetic material is used in combination with another material, i.e. when a liner and socket material are used together. However, it should be noted that for the same, the steady-state temperature at the liner interface of the combination *T*_2ss_ is utilised and not *T*_3ss_. This is because the mathematical model that is used for non-invasive measurement predicts the residual limb temperature by measuring the liner temperature, and hence the thermal time constant at the liner interface of the combination is of interest. Hence, the generation of an exponentially decaying data is done by subtracting the liner temperature at ambient time *T*_2_ from *T*_2ss_.

As in step 3, ln[*x*(*T*_2ss_) − *x*(*T*_2_)] is plotted with respect to time and the slope of line is indicative of the thermal time constant of the liner-socket material combination measured at the liner interface. Figs. 4*a* and 5*a* illustrate the temperature profile of the polyurethane liner when used individually and in combination (with thermosetting socket material), respectively, which is then utilised to compute the thermal time constant by the method described above.

Thermal time constant is essentially the same for all starting temperatures. The process to determine the thermal time constant as described above is repeated for different heat source temperatures (from 30 to 40°C, with increasing intervals of 2°C). This is to confirm the accuracy of time constant value of the liner when used individually or in combination of a socket material and rule out any experimental errors. Hence the value of *τ* so computed when the polyurethane liner is used individually is 2.8 min, whereas when it is tested in combination with a thermosetting socket material it is ∼5.4 min.

The thermal time constants for the prosthetic materials (when used individually) as listed in Table 2 were also determined using the procedure described. The results of the same as in Table 3 do confirm to [7] and suggest that the prosthetic materials can act as a barrier to conductive heat transfer due to their low thermal conductivity or high thermal time constants. It can be also seen from the results that there is substantial variation in the time constants of liner materials, whereas the prosthetic socket materials have similar time constants. Thus, we can conclude that the selection/combination of prosthetic materials have a considerable impact on the residual limb skin temperature as they can produce different thermal environments. This can be further seen in Table 3 where the time constants of some of the widely used liner-socket combinations are detailed.

**Table 2:** Time constants for liner and socket materials when evaluated individually

Name	Time constant *τ*, min
Alpha Locking (liner)	3.6
Iceross Comfort (liner)	3.1
Iceross Original (liner)	2.6
OttoBock Technogel (liner)	2.8
Pe-lite (liner)	1.6
stump sock	0.6
thermoplastic (socket material)	4.0
thermosetting lay-up (socket material)	4.1
carbon fibre lay-up (socket material)	4.5

**Table 3:** Time constants of the liner and socket materials when evaluated in a combination

Combination of prosthetic materials	Time constant *τ*, min
OttoBock Technogel (polyurethane liner – 6 mm) with	5.4
thermosetting lay-up (socket material – 4 mm)	
Iceross Comfort (silicone liner – 6 mm) with	5.5
carbon fibre lay-up (socket material – 4.8 mm)	
Iceross Original (silicone liner – 3 mm) with	4.1
carbon fibre lay-up (socket material – 4.8 mm)	
Iceross Comfort (silicone liner – 6 mm) with	5.8
thermosetting lay-up (socket material – 4 mm)	
Alpha Locking (co-polymer liner – 6 mm) with	6.2
carbon fibre lay-up (socket material – 4.8 mm)	
Pe-lite (closed cell foam – 5 mm) with	6.7
thermoplastic (socket material – 4.7 mm)	

## 5 Gaussian process modelling

In our study, two transtibial traumatic amputees with the details listed in Table 4 were recruited. The details of the 35 min clinical trial were similar to as described in [13] – donning/resting for 10 min, walking on the treadmill for 10 min and final resting for 15 min. To monitor and record the residual limb and liner-socket temperatures, four *K*-type thermocouples via a data logger (type HH1384; Omega Engineering) were used. One thermocouple was taped on the lateral side of the limb and the other on the medial. The other two thermocouples were taped on the corresponding point on the liner-socket interface. Data from four channels was recorded at 0.5 Hz at a defined ambient temperature (dataset A). This was repeated again after two months to confirm the influence of ambient temperature on the residual limb skin temperature (dataset B). The temperature profiles of the liner and the residual limb skin were recorded in a climate controlled chamber with zero wind velocity and 40% humidity levels for ambient temperatures of 10°C, and then the same protocol was repeated for 15, 20 and 25°C. The results for both the subjects indicated that for any given ambient temperature, the liner temperature profile follows that of the in-socket residual limb temperature. This suggested a possibility to apply supervised machine learning algorithms to model the residual limb temperature of the amputee as a function of liner temperature. Time averaging of 5 s is done on the recorded data to help in identifying the trend better and smooth out the fluctuations. Since, the temperature profiles of the residual limb are almost similar for the ambient temperature pairs of 10°C, 15°C and 20°C, 25°C, the individual predictive model for both the subjects at ambient temperatures of 10 and 25°C are only discussed in this Letter. Hence experimentation for each amputee subject was used to develop individual predictive models using GPML [13].

**Table 4:** Details of the amputee subjects

Amputee	Age, years	Weight, kg	Details of the prosthesis
subject 1	68	70	OttoBock Technogel (polyurethane liner – 6 mm) with thermosetting lay-up (socket material – 4 mm)
subject 2	63	69.8	Pe-lite (closed cell foam – 5 mm) with thermoplastic (socket material – 4.7 mm)

The GPML model aims to determine the liner temperature as a function of skin temperature and use it for predictive analysis. Processing was performed with custom developed software (using MATLAB^®^, Mathworks). The model designed takes the liner temperature as the input *x* and the predicted output is the residual limb skin temperature *y*. The Gaussian process technique is a supervised learning algorithm which infers a continuous function *f*(*x*) from a training set of input–output pairs. The key assumption in this method is this collection of random variables, any finite number of which have joint Gaussian distributions [14]. Therefore, it could be totally specified by the mean and covariance function. A Gaussian process model can be used as a prior probability distribution over functions in Bayesian inference. This enables deducing the hyperparameters for the model which are an indication of the precision and relevance of the input parameters for predicting the output. Thus, the aim in Gaussian process modelling is to select the model parameters for which the probability of the training data is maximised [14, 15]. This can be implemented by using the Bayes’ theorem.

For *N* pairs of input–output (*x*_*N*_, *y*_*N*_), the Gaussian model is defined by *N*-dimensional covariance matrix ***C****_N_* which indicates the degree of closeness of outputs for varying inputs. Each element of ***C****_N_* is defined by covariance function *C*_f_, which is a function of inputs and hyperparameters [14, 15]. For the element *ij* in covariance matrix*C*_*ij*_ = *C*_f_(x_i_, x_j_, Θ). The covariance function can be defined by user depending on the nature of the input–output response. The squared exponential covariance function was the best fit for our study

(10) $${C_{\rm f}} = {\theta _1}{{\rm e}^{ - \left({{{{{\lpar {x_i} - {x_j}\rpar }^2}} / {2{l^2}}}} \right)}} + \sigma _n^2 {\delta _{ij}}\eqno\lpar 10\rpar $$

**Fig. 6:**
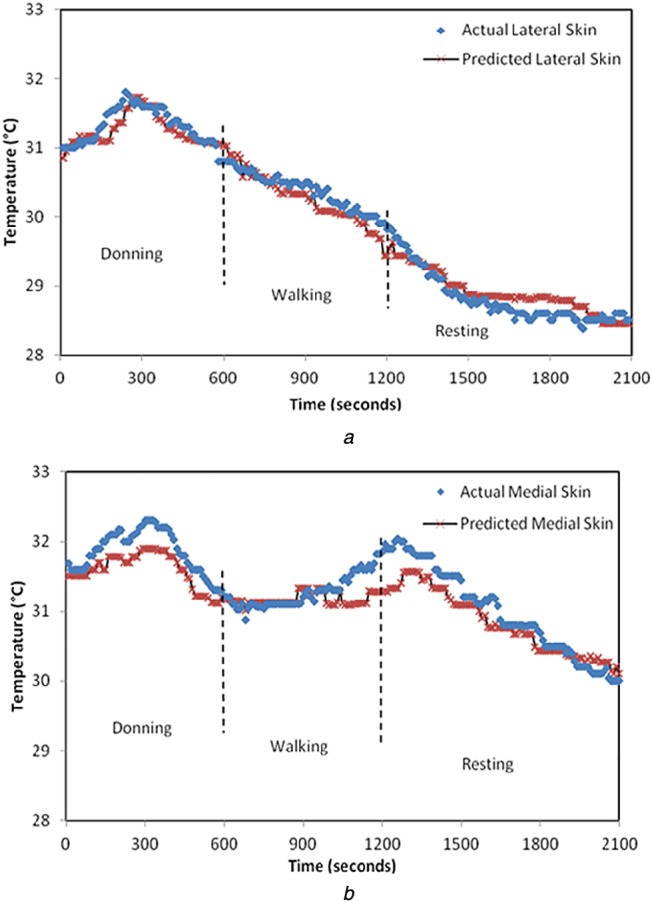
Predicted and actual residual limb temperature for Subject 1 at ambient temperature of 10°C is shown for *a* Lateral side *b* Medial side

**Fig. 7:**
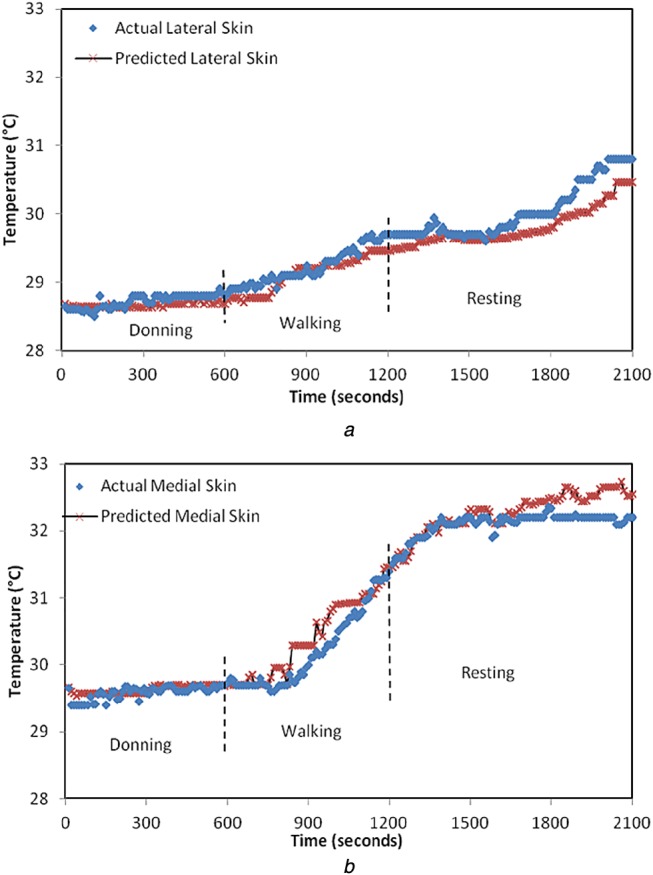
Predicted and actual residual limb temperature for Subject 1 at ambient temperature of 25°C is shown for *a* Lateral side *b* Medial side

**Fig. 8:**
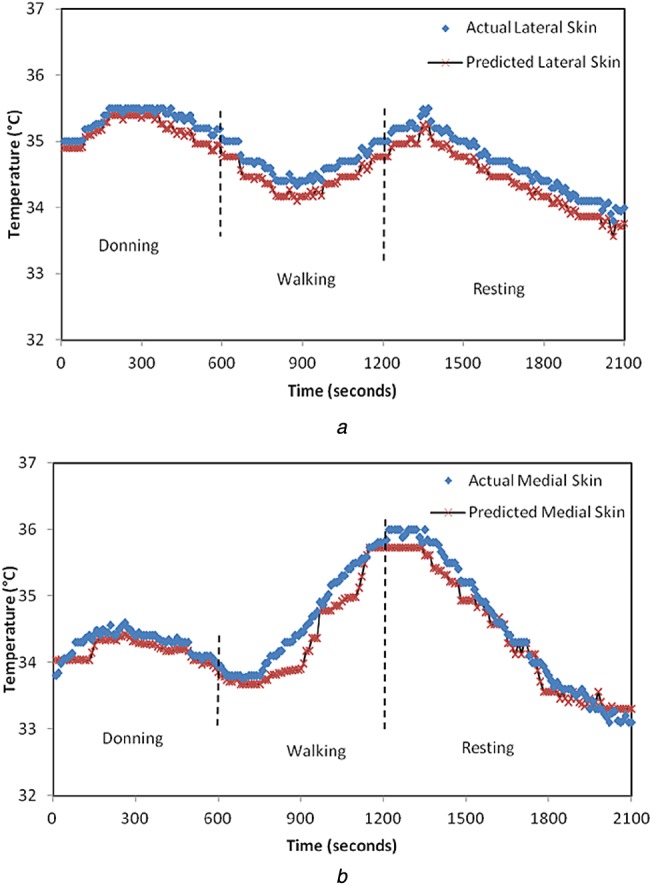
Predicted and actual residual limb temperature for Subject 2 at ambient temperature of 10°C is shown for *a* Lateral side *b* Medial side

**Fig. 9:**
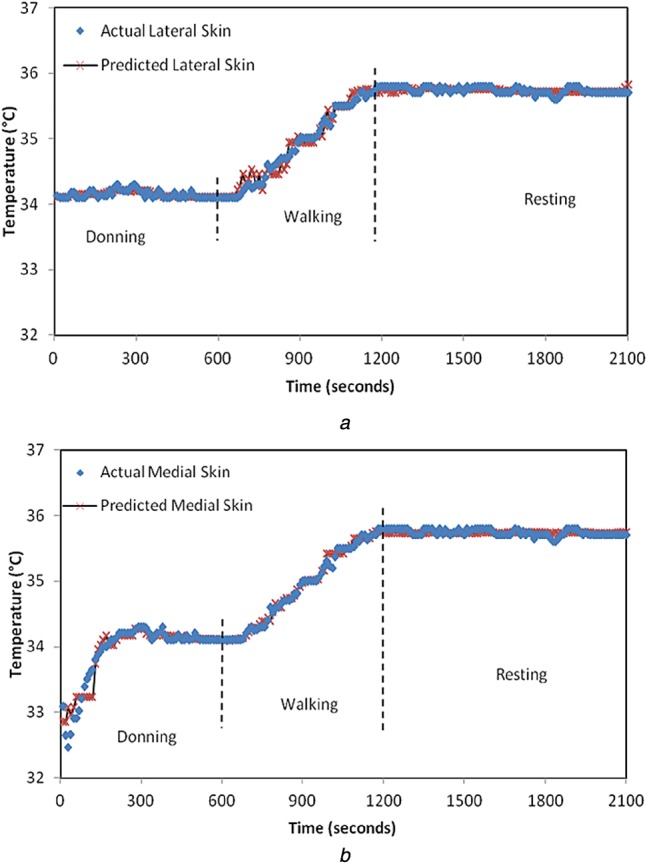
Predicted and actual residual limb temperature for Subject 2 at ambient temperature of 25°C is shown for *a* Lateral side *b* Medial side

In (10), the set of hyperparameters are Θ = {*θ*_1_, *l*, *σ*_*n*_} and *δ*_*ij*_ which is a delta function whose value is zero for all *i≠j*. The length scale *l* for an input parameter indicates how much the output will vary relative to changes in an input. This, if correlated to the heat transfer in prosthetic material, implies that the thermal time constant is a measure of how quickly the in-socket temperature changes with the change in residual limb temperature. Hence, if the length scale in the covariance function is set to the thermal time constant of the materials used in the prosthetic limb, it would optimise the Gaussian process model and generalise it for amputee subjects with similar prosthesis setup. For Subject 1, the value of length scale is specified as 5.4, which is equal to the time constant of the materials used in his prosthesis setup. Similarly, for Subject 2, the length scale is defined to be 6.7. When *l* is specified, less number of iterations are required for the computation of other hyperparameters. This in turn minimises the log marginal likelihood function to give the best predictions. The results from this model (with the length scale equal to the thermal time constant) lie in 95% confidence interval which translates to an accuracy of ±0.5°C. With the introduction of the thermal time constant as the length scale in the covariance function, the physical properties of the prosthetic material are accounted for in the model as opposed to in [13] which is a purely empirical model. The actual skin temperature of Subject 1 obtained by the Gaussian predictive model is shown in Figs. 6 and 7 for two very different ambient temperatures of 10 and 25°C, respectively. Similarly, Figs. 8 and 9 represent the actual skin temperature of Subject 2 obtained by the Gaussian predictive model. From Figs. 6 to 9, it can be seen the predicted skin temperature (at lateral and medial sides) for both the subjects follows the corresponding actual skin temperature with an accuracy of ±0.5°C. This is a significant improvement as compared with the accuracy of the existing model which was ±0.8°C.

## 6 Conclusion

The accuracy of the model developed to non-invasively monitor the residual limb temperature of an amputee has been improved to ±0.5°C. It is indicated from the study that the residual limb temperature depends on the ambient temperature and the activity level of the subject. Also, a major factor is the thermal time constant of the prosthetic materials used. Owing to the low thermal conductivity of the prosthetic materials, it can restrict the heat transfer from the residual limb and create a warm microenvironment within the prosthesis. Hence, it becomes all the more imperative to build in the existing GPML model the thermal time constant so obtained from the thermal studies. It was also found that different prosthetic materials transfer heat logarithmically at different rates and that they also transfer different amounts of heat. Further to this it was found that placing these materials in combinations slowed the rate of heat transfer and also decreased the maximum amount of heat transferring through the materials.

Thus, this Letter highlights the relevance of thermal time constant of prosthetic materials in Gaussian processes technique which would be useful in addressing the challenge of non-invasively monitoring the residual limb skin temperature. With the introduction of thermal time constant in the model, the accuracy increases, thereby making predictions more reliable. Also, this approach is quite useful in extending the model to a wider amputee population to define a generic behaviour. Future scope of the work includes studying the interplay between temperatures and sweating response in prosthesis of amputees with different pathologies.
